# A quantitative model of the phytochrome-PIF light signalling initiating chloroplast development

**DOI:** 10.1038/s41598-017-13473-2

**Published:** 2017-10-24

**Authors:** Carole Dubreuil, Yan Ji, Åsa Strand, Andreas Grönlund

**Affiliations:** Umeå Plant Science Centre, Department of Plant Physiology, Umeå University, SE-90187, Umeå, Sweden

## Abstract

The components required for photosynthesis are encoded in two separate genomes, the nuclear and the plastid. To address how synchronization of the two genomes involved can be attained in early light-signalling during chloroplast development we have formulated and experimentally tested a mathematical model simulating light sensing and the following signalling response. The model includes phytochrome B (PhyB), the phytochrome interacting factor 3 (PIF3) and putative regulatory targets of PIF3. Closed expressions of the phyB and PIF3 concentrations after light exposure are derived, which capture the relevant timescales in the response of genes regulated by PIF3. Sequence analysis demonstrated that the promoters of the nuclear genes encoding sigma factors (SIGs) and polymerase-associated proteins (PAPs) required for expression of plastid encoded genes, contain the cis-elements for binding of PIF3. The model suggests a direct link between light inputs via PhyB-PIF3 to the plastid transcription machinery and control over the expression of photosynthesis components both in the nucleus and in the plastids. Using a pluripotent Arabidopsis cell culture in which chloroplasts develop from undifferentiated proplastids following exposure to light, we could experimentally verify that the expression of SIGs and PAPs in response to light follow the calculated expression of a PhyB-PIF3 regulated gene.

## Introduction

Mathematical modelling of physicochemical processes and the following analysis of such models drives new hypotheses which can be experimentally tested and falsified to step-by-step improve our understanding of cellular processes. Molecular interactions can be identified using experimental and statistical tools and temporal aspects of cellular processes can be modelled as dynamical systems, describing the kinetics of birth, death and interaction of molecules^1–3^. Frequently, biochemical reaction networks of higher organisms are modelled using systems of differential equations describing the mass action kinetic of the individual concentrations. However, some properties of cellular processes may only be captured in more detailed models that include individual binding events^4^, delayed reactions^5,6^, or spatial and stochastic properties^7^ and possibly also additional biophysical aspects of the studied system.

In eukaryotic cells photosynthesis occurs in the chloroplasts. The chloroplast has its own genome but the photosynthetic machinery is built using proteins encoded both in the nucleus and in the plastids. The establishment of photosynthesis during the greening process therefore requires a coordination of the activities of these two distinct genomes. Plastid genes are transcribed by two different RNA polymerases; a nuclear encoded RNA polymerase (NEP) and a plastid encoded RNA polymerase (PEP), which is a eubacterial-type multi-subunit enzyme. PEP represents the major transcription machinery in mature chloroplasts and over 80% of all primary plastid transcripts are transcribed by PEP^8^. The core components of PEP are encoded in the plastids but the PEP complex rely on the nuclear encoded sigma factors (SIGs) to bind and initiate transcription. In addition to the SIGs, PEP is only active when forming a complex with polymerase associated components (PAPs)^9,10^, which are also encoded in the nucleus and transported to the plastids. The nuclear encoded SIGs and PAPs provide a remote control mechanism of plastid gene expression where the activity in the plastids can be controlled by the nucleus and the activities of the two genomes coordinated.

Phytochromes (Phy) are light sensing molecules interchanging between two different states depending on the light composition and intensity. The inactive form (Pr) absorbs red light and is then converted to an active form (Pfr). Pfr is converted back to Pr in the presence of far-red light. After a long period of darkness, the inactive state is dominating due to a dark reversion rate and in light an equilibrium of Pr to Pfr is rapidly attained. The distribution between the forms depends on the red to far-red light intensity ratio. More red light compared to far-red light gives more Pfr and vice versa. The phytochromes give plants a molecular mechanism to collect and transfer information of the prevailing light conditions. Through the action of the photoreceptors, a large reorganization of the nuclear transcriptional program is manifested as a response to light^11^.

Phytochrome interacting factor 3 (PIF3) is a basic helix-loop-helix transcription factor that binds to *cis*-regulatory elements in the promoter of various genes^12^. Binding of the active form of phytochrome B (PhyB) to PIF3 induce phosphorylation and subsequent degradation of PIF3 by 26 S proteasomes. In this way PhyB mediates information of the surrounding light to the expression level of genes regulated by PIF3. It is shown that PIF3 is a repressor of chloroplast development in the dark^13^. The rapid degradation of PIF3 imply that genes repressed by PIF3 are activated in response to a shift from dark to light^14^ and that these genes play a key role in the initiation of photomorphogenic development^15^.

Chloroplast biogenesis is closely interconnected with photomorphogenesis, which complicates the analysis of chloroplast development *per se* and many of the mutations described are seedling- or embryo-lethal. We have generated a pluripotent inducible cell line from Arabidopsis^16^, which in contrast to other cell cultures is not constitutively green^17^. Our cell line can be propagated in the dark and after light exposure gradually induces a greening process where, just like in leaves, proplastids are transferred directly to photosynthetic active chloroplasts without the intermediate etioplast form^18^.

Many processes in plants are light regulated and by formulating a mathematical model of the initial light response triggered by phyB, PIF3 and gene targets repressed by PIF3 in the dark we have explored the regulatory kinetics of such processes. By performing promoter sequence analysis we could demonstrate that *SIG*s and *PAP*s could be putative target genes regulated by PIF3. We therefore tested the calculated response from the dynamic model experimentally by determining the expression profile in response to light for SIGs and PAPs in our single-cell experimental system where chloroplasts are developed upon shift from dark to light.

## Results

### A single cell Arabidopsis experimental system

**Figure 1:**
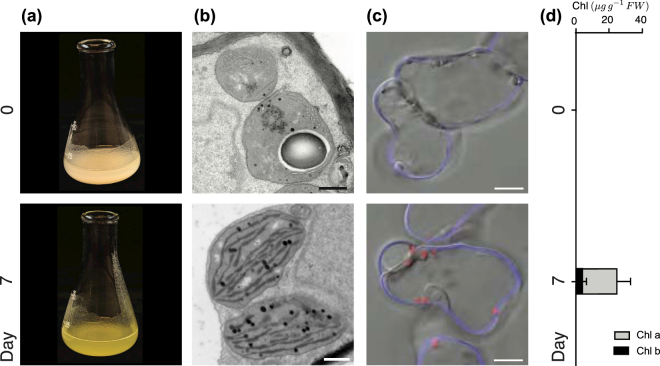
Chloroplast biogenesis induced by light in Arabidopsis meristematic cell culture. The cells show mature chloroplasts after 7-days of continuous light which is demonstrated here by: (**a**) The greening of the cell culture. (**b**) Ultrastructure of pro-plastids developing into chloroplasts. Representative electron microscopy images were chosen from at least two experiments and time points. Scale bar: 0.5 *μ*m. (**c**) Confocal microscopy of chlorophyll accumulation visualized in red by its autofluorescence. The cell wall is stained with calcofluor-white in blue. Scale bar: 10 *μ*m. (**d**) Chlorophyll content *μg* per gram fresh weight.

In darkness, the cells obtain their energy from sucrose in the medium (see Methods section), but after light exposure the cells propagate without any further addition of sucrose. The chloroplast differentiation in the light is reflected by the greening of the cells Fig. 1(a) and the increase in chlorophyll content Fig. 1(c) and (d). After 7 days in light the chloroplasts have a well-developed structure with numerous intergranal thylakoids Fig. 1(b), similar to what is observed in Arabidopsis seedlings^19^.Taken together, these results demonstrate that this pluripotent inducible cell culture system behaves in many ways like leaf mesophyll cells and can develop functional chloroplasts on demand. Thus, our cells mimic the process of chloroplast development in developing leaves and provides an experimental system with strict control of the initiation of chloroplast development. Moreover, in contrast to whole plants, the single-cell system makes it possible to observe synchronous development of many cells in parallel making it possible to investigate the temporal properties of the regulatory mechanisms behind the transition from proplastids to chloroplasts.

### PhyB-PIF3 involvement in SIG and PAP light response

**Figure 2:**
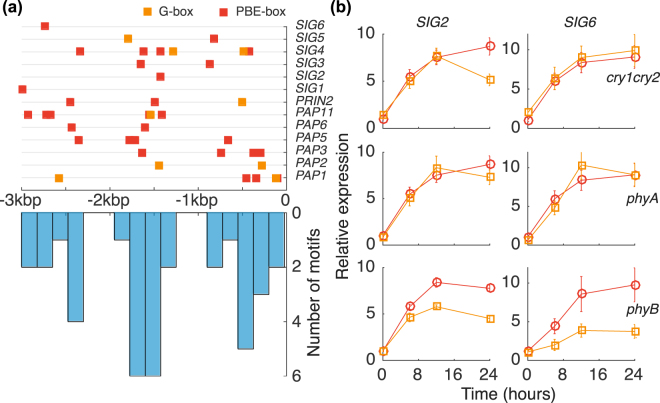
PIF3 binding motif localization upstream of *PAP* and *SIG* genes and expression of *SIG* in photoreceptor mutants. In (**a**) individual positions of G-box (CACGTG) and PBE-box (CACATG) binding motifs 3 kb upstream from ATG (upper) and a histogram of the motif positions (lower). (**b**) Expression after light induction of *SIG2* (left column) and *SIG6* (right column) in WT (red circle symbols) and *cry1cry2*, *phyA* and *phyB* mutants (orange square symbols). 5-day old dark grown seedlings were exposed to light and sampled for RNA-extraction.

Several of the components required for transcription in the plastids are nucleus-encoded such as the sigma factors (SIG) of PEP^20^ and the PAPs^21^. PLASTID REDOX INSENSITIVE 2 (PRIN2) is a protein that just like the PAPs are encoded in the nucleus and required for full PEP activity^22^. To understand the mechanism behind the regulation of these components we analysed their promoter sequences for known regulatory motifs of light signalling. Zhang *et al*.^12^, performed integrated ChIP-seq and RNA-seq analyses and showed that PIF3 transcriptional regulation is exerted by sequence-specific binding to the G-box (CACGTG) or the PBE-box (CACATG) motifs in the target promoters of genes in *Arabidopsis*. The identified PIF3-binding sites were within 3 kb of the transcription start site for 88% of the genes^12^. To test if the G-box or PBE-box motifs were associated with *PAP*, *PRIN2* and *SIG* genes we performed MEME-FIMO analysis of all the *SIG* genes (*SIG1-6*) and the genes encoding proteins defined as true *PAP*s using a 3 kb restriction. The MEME analysis demonstrated that the G-box was identified for *PAP1*, *PAP2*, *PAP11* and *PRIN2* and also *SIG4* and *SIG5*, Fig. 2(a) and Supplementary Table S1. The PBE-box was found in the promoters of all the *SIG*s (SIG1-6) and *PAP1*, *PAP3*, *PAP5*, *PAP6*, *PAP11*, *FLN2* and *PRIN2*, Fig. 2(a) and Supplementary Table S2. The remaining *PAP*s all contain a PBE core element (Supplementary Table S3). Moreover, the identified motifs were not randomly distributed upstream of the ATG start codon but rather clustered to three distinct regions, see Fig. 2(a), suggesting that the motifs have functional role. Mutant analyses have shown that *SIG2* and *SIG6* are essential during early light response and seedling development^23^. In contrast to mutations that affect the other SIGs, the *sig2* and *sig6* seedlings are pale and accumulated less chlorophyll. Moreover, PhyB is shown to induce *SIG2* expression^24^ and control *SIG6* during photomorphogenesis^25^. Thus, our focus for the experimental work was on *SIG2* and *SIG6*. To further investigate the involvement of PhyB-PIF3 we used *cry1cry2*, *phyA* and *phyB* mutants. The expression of *SIG2* and *SIG6* after light induction is seen in Fig. 2(b). Of the three mutants investigated, only *phyB* mutant deviate from wild-type expression for both *SIG2* and *SIG6* throughout the initial light response. Thus, the identified PIF3 binding motifs, the non-random motif distribution and the determined SIG expression in *cry1cry2*, *phyA* and *phyB* suggests an involvement of PhyB-PIF3 in the regulation of the components required for PEP activity and activation of transcription in the chloroplast.

### The Phy-PIF response

**Figure 3:**
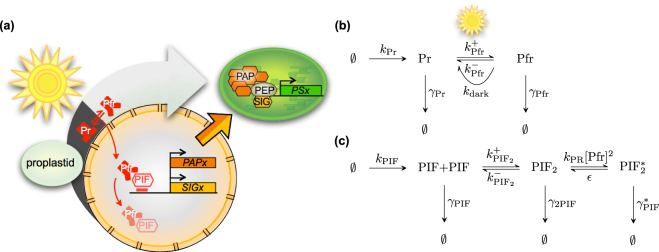
Model of light signaling of phytochrome B and PIF3. In (**a**) an illustration displaying the initiation of plastid gene expression by regulating *PAP* and *SIG* gene expression. Upon light induction phytochrome B transform to the active Pfr form which induce degradation of PIF3 and and thereby activating PIF3 repressed genes. (**b**) The reaction scheme of phytochrome production, decay and conversion between Pr and Pfr. Upon light induction, Pr is converted to Pfr and the ratio depends on the spectral composition of the light. In darkness, the dark reversion rate convert Pfr to the Pr form. The spectral composition of our light source have a red/far-red ratio of 10:1 making the Pfr form dominate in light. (**c**) The chemical reaction scheme of PIF3 in presence (and absence) of Pfr. In presence of Pfr, PIF3 is rapidly phosphorylated and degraded. This is captured in the model if the backward, un-phosphorylation, rate *ε* is small compared to the phosphorylation rate *k*_PR_[Pfr]^2^ and the decay rate ${\gamma }_{{\rm{PIF}}}^{\ast }$.

The phytochromes can exist in different forms and more or less detailed models are developed to capture the the behaviour of the different states phytochromes can form see e.g.^26–28^. The reactions are illustrated in Fig. 3(a) and (b) and we follow the simplification made in^26^ and consider PhyB to be synthesised with the rate *k*_*Pr*_ to its Pr form. Pr have decay rate constant *γ*_Pr_ and can also transition to its (active) Pfr form, a reaction with rate constant ${k}_{{\rm{P}}{\rm{f}}{\rm{r}}}^{+}$. The active Pfr-form have decay rate constant *γ*_Pfr_ and can transition back to the Pr form, in light given by the rate constant ${k}_{{\rm{P}}{\rm{f}}{\rm{r}}}^{-}$ and in darkness by the dark reversion rate *k*_dark_. The ratio of Pr to Pfr phytochrome concentration is dictated by the equilibrium constant ${K}_{{\rm{P}}{\rm{f}}{\rm{r}}}={k}_{{\rm{P}}{\rm{f}}{\rm{r}}}^{+}/({k}_{{\rm{P}}{\rm{f}}{\rm{r}}}^{-}+{k}_{{\rm{d}}{\rm{a}}{\rm{r}}{\rm{k}}})$. After long periods of darkness most of the phytochromes are in the Pr form due to the dark reversion rate and when there is light phytochromes are equilibrated in a ratio Pfr/Pr depending on the spectral content of the light. The equilibrium constant *K*_Pfr_ therefore serve as the light sensor of the reaction system. The reactions in Fig. 3(b) can be translated to the following system of differential equations, describing the rate change of the concentrations of the two forms Pr and Pfr,

1 $$\begin{array}{rcl}\frac{d[\Pr ]}{dt} & = & {k}_{{\rm{\Pr }}}{+(k}_{{\rm{Pfr}}}^{-}{+k}_{{\rm{dark}}}{)[\text{Pfr}]-(k}_{{\rm{Pfr}}}^{+}{+{\rm{\gamma }}}_{{\rm{\Pr }}})[\Pr ]\\ \frac{d[\text{Pfr}]}{dt} & = & {k}_{{\rm{Pfr}}}^{+}{[\Pr ]-(k}_{{\rm{Pfr}}}^{-}{+k}_{{\rm{dark}}}{+{\rm{\gamma }}}_{{\rm{Pfr}}})[\text{Pfr}]\end{array}$$

The total concentration of phytochrome B is [PhyB] = [Pr] + [Pfr]. Assume that the two forms Pr and Pfr equilibrates fast such that [Pfr] = *K*_Pfr_[Pr]. The total phytochrome concentration will then evolve according to the following differential equation

2 $$\frac{d[\text{PhyB}]}{dt}={{\rm{\gamma }}}_{{\rm{\Pr }}}(\frac{{k}_{{\rm{\Pr }}}}{{{\rm{\gamma }}}_{{\rm{\Pr }}}}-(\frac{1+{{\rm{\Gamma }}}_{{\rm{Pfr}}}{K}_{{\rm{Pfr}}}}{1+{K}_{{\rm{Pfr}}}})[\text{PhyB}])$$

where Γ_Pfr_ = *γ*_Pfr_/*γ*_Pr_ is ratio of the decay rate constant of the short-lived Pfr-form to the long lived Pr-form. In dark, all (or at least most) phytochromes are in the the Pr form due to the dark reversion rate *k*_*dark*_, giving *K*_*Pfr*_ ≈ 0. The stationary concentration in darkness, [PhyB_s_]_D_, is obtained by letting *d*[PhyB]/*dt* = 0,

3 $${{[\text{PhyB}}_{s}]}_{{\rm{D}}}=\frac{{k}_{{\rm{\Pr }}}}{{\gamma }_{{\rm{\Pr }}}}$$

we rescale the phytochrome with the dark stationary concentration, which will be the initial concentration, such that $[{\rm{P}}{\rm{h}}{\rm{y}}{\rm{B}}]\mapsto [{\rm{P}}{\rm{h}}{\rm{y}}{\rm{B}}]/[{\text{PhyB}}_{s}{]}_{{\rm{D}}}$. At time *t* ≥ 0 light is induced and the evolution of the scaled total concentration will evolve according to the following initial value problem

4 $$\{\begin{array}{ll}{[{\rm{PhyB}}]}_{t=0} & =\,1\\ \frac{d[{\rm{PhyB}}]}{dt} & =\,{\gamma }_{{\rm{\Pr }}}(1-{Q}_{{\rm{Pfr}}}[{\rm{PhyB}}])\end{array}$$

where *Q*_Pfr_ = (1 + Γ_Pfr_ *K*_Pfr_)/(1 + *K*_Pfr_). The solution of (4) can be obtained with the use of integrating factor. The solution is given by

5 $$[\text{PhyB}](t)={Q}_{{\rm{Pfr}}}^{-1}+(1\,-\,{Q}_{{\rm{Pfr}}}^{-1}{)e}^{-{Q}_{{\rm{Pfr}}}{\gamma }_{{\rm{\Pr }}}t}$$

Now, we need to calculate the Pfr concentration, since the Pfr-form is the active form that induce degradation of PIF3. Assuming fast equilibration, we have

6 $$[\text{Pfr}](t)={R}_{{\rm{Pfr}}}[\text{PhyB}](t)$$

where *R*_Pfr_ = *K*_Pfr_/(1 + *K*_Pfr_) is the fraction of Pfr in total Phy concentration. Since Pfr absorb some red light there is still a fraction of about 13% Pr in red light, making *R*_Pfr_ ≈ 0.87 and thus *K*_Pfr_ ≈ 7 in red light. The half-life of PhyB in red light is observed to be approximately 8 hours^29^ making *Q*_Pfr_ *γ*_Pr_ ≈ 3log(2) days^−1^. After light is induced, the phytochromes switch to the Pfr state and interacts with PIF. The interaction with Pfr induces a rapid phosphorylation of PIF which makes it accessible for degradation by 26 S proteasomes. The reaction scheme of PIF is displayed in Fig. 3c.

The rate constants are given by; the phosphorylation rate constant *k*_PR_, the un-phosphorylation rate constant *ε*, the phosphorylated state of PIF [Pfr]^*^ and the proteosomal degradation rate constant *γ* ^*^. It is shown that the gene expression of PIF is not changing much in shift from dark to light and that PIF protein concentration is predominantly regulated post-translationally by degradation mediated by active Phy^30,31^. We therefore make the simplification of having a constitutive, non-changing, transcription rate of PIF in the model. The rate of change in the PIF and PIF ^*^ concentrations can be summarised by the three following differential equations.

7 $$\begin{array}{rcl}\frac{d[\text{PIF}]}{dt} & = & {k}_{{\rm{PIF}}}-{\gamma }_{{\rm{PIF}}}[\text{PIF}]-{k}_{{{\rm{PIF}}}_{2}}^{+}{[\text{PIF}]}^{2}+{k}_{{{\rm{PIF}}}_{2}}^{-}{[\text{PIF}}_{2}]\\ \frac{{d[\text{PIF}}_{2}]}{dt} & = & {k}_{{{\rm{PIF}}}_{2}}^{+}{[\text{PIF}]}^{2}-{k}_{{{\rm{PIF}}}_{2}}^{-}{[\text{PIF}}_{2}]-{k}_{{\rm{PR}}}{K}_{{\rm{\dim }}}^{{\rm{Pfr}}}{[\text{Pfr}]}^{2}{[\text{PIF}}_{2}]\\  &  & {-\gamma }_{{\rm{2PIF}}}{[\text{PIF}}_{2}]+\varepsilon {[\text{PIF}}_{2}^{\ast }]\\ \frac{d{[\text{PIF}}_{2}^{\ast }]}{dt} & = & {k}_{{\rm{PR}}}{[\text{Pfr}]}^{2}{[\text{PIF}}_{2}]-\varepsilon {[\text{PIF}}_{2}^{\ast }]-{\gamma }_{{\rm{PIF}}}^{\ast }{[\text{PIF}}_{2}^{\ast }]\end{array}$$

where we have assumed that Pfr phosphorylates PIF as dimers, giving a squared dependence of the Pfr concentration to the phosphorylation rate, with ${K}_{{\rm{d}}{\rm{i}}{\rm{m}}}^{{\rm{P}}{\rm{f}}{\rm{r}}}=[{{\rm{P}}{\rm{f}}{\rm{r}}}_{2}]/[{\rm{P}}{\rm{f}}{\rm{r}}{]}^{2}$. It should also be noted that Pfr may form hetero-dimers with Pr and can form nuclear bodies^27,28^, which imply that the degradation rate of PIF3 will depend upon the concentration of the state (or states) that induce degradation of PIF3. If we assume that the equilibration between all PhyB states in light is fast we can view ${K}_{{\rm{\dim }}}^{{\rm{Pfr}}}$ as the effective equilibrium constant of the Pfr dimer state(s) that induce degradation of PIF3. To proceed we note that the phosphorylation of PIF is experimentally observed to be fast and that the PIFs are rapidly removed in presence of Pfr dimers, which we obtain if *ε* is small compared to *k*_PR_[Pfr]^2^ and ${\gamma }_{2}^{\ast }$, i.e. PIF is rapidly phosphorylated and degraded and the backward reaction can practically be ignored. Assuming also a quasi steady state $\tfrac{d[{{\rm{PIF}}}_{2}]}{dt}=0$, ${K}_{\dim }^{{\rm{P}}{\rm{I}}{\rm{F}}}={k}_{{{\rm{P}}{\rm{I}}{\rm{F}}}_{2}}^{+}/{k}_{{{\rm{P}}{\rm{I}}{\rm{F}}}_{2}}^{-}=[{{\rm{P}}{\rm{I}}{\rm{F}}}_{2}]/{[{\rm{P}}{\rm{I}}{\rm{F}}]}^{2}$ and that the decay rate of (non-phosphorylated) dimers *γ*_2*PIF*_ is small we obtain

8 $$\frac{d[\text{PIF}]}{dt}={k}_{{\rm{PIF}}}-{\gamma }_{{\rm{PIF}}}[\text{PIF}]-{k}_{{\rm{PR}}}{K}_{{\rm{\dim }}}^{{\rm{PIF}}}{K}_{{\rm{\dim }}}^{{\rm{Pfr}}}{[\text{Pfr}]}^{2}{[\text{PIF}]}^{2}$$

Re-arranging the terms gives the relative to dark concentration level of PIF, $[{\rm{P}}{\rm{I}}{\rm{F}}]\mapsto [{\rm{P}}{\rm{I}}{\rm{F}}]/[{\rm{P}}{\rm{I}}{\rm{F}}{]}_{{\rm{D}}}$ and expressing Pfr in terms of PhyB levels, eqn. (6), gives

9 $$\frac{d[\text{PIF}]}{dt}={\gamma }_{{\rm{PIF}}}(1-[\text{PIF}]-{R}_{{\rm{PP}}}{[\text{PhyB}]}^{2}{[\text{PIF}]}^{2})$$

where ${R}_{{\rm{PP}}}={k}_{{\rm{PR}}}{K}_{{\rm{\dim }}}^{{\rm{PIF}}}{K}_{{\rm{\dim }}}^{{\rm{Pfr}}}{R}_{{\rm{Pfr}}}^{2}{[{\rm{PIF}}]}_{{\rm{D}}}/{\gamma }_{{\rm{PIF}}}$, which measures the interaction strength between PhyB and PIF3, since *R*_Pfr_ is the fraction of Pfr in PhyB and ${K}_{{\rm{\dim }}}^{{\rm{Pfr}}}$ the equilibrium level of Pfr dimers phosphorylating PIF3 with rate *k*_PR_. If the PhyB-PIF3 interaction strength is zero *R*_PP_ = 0 we have [PIF] = 1, the dark initial concentration. At the light switch where a large fraction of Pr transition to Pfr, the decay of PIF3 is fast (half-life ≈15 min) compared to the change in PhyB concentration (half-life ≈  8 hours) and we can for short time-scales treat PhyB as constant. Thus, we assume PhyB ≈ 1 during the initial equilibration of PIF to the new Pfr level after light-induction. Under such assumptions eqn. (9) can be approximated as,

10 $$\begin{array}{ll}\frac{d{[{\rm{PIF}}]}_{{\rm{fast}}}}{dt} & \approx -{\gamma }_{{\rm{PIF}}}{R}_{{\rm{PP}}}{[{\rm{PIF}}]}^{2}\\ \Rightarrow {[{\rm{PIF}}]}_{{\rm{fast}}}(t) & \approx \frac{1}{C+{\gamma }_{{\rm{PIF}}}{R}_{{\rm{PP}}}t}\end{array}$$

where the integration constant *C* will be determined from the initial condition. If the PIF concentration equilibrates quickly with the slowly varying Pfr concentration, the slowly varying solution of PIF can be obtained by letting the left-hand side of eqn. (9) equal to zero, and solving the right-hand side w.r.p to PIF concentration,

11 $${[\text{PIF}]}_{{\rm{s}}{\rm{l}}{\rm{o}}{\rm{w}}}(t)\approx \frac{1}{2{R}_{{\rm{P}}{\rm{P}}}{[\text{PhyB}]}^{2}(t)}(\sqrt{1+4{R}_{{\rm{P}}{\rm{P}}}{[\text{PhyB}]}^{2}(t)}-1)$$

Combining (10) and (11) with the initial condition that [PIF](0) = 1, assuming $4{R}_{{\rm{PP}}}{Q}_{{\rm{Pfr}}}^{-1}\gg 1$ and omitting the slowly varying time dependence in the square root we obtain

12 $$[\text{PIF}](t)\approx \frac{1}{1+{\gamma }_{{\rm{P}}{\rm{I}}{\rm{F}}}{R}_{{\rm{P}}{\rm{P}}}{\rm{t}}}+\frac{{Q}_{{\rm{P}}{\rm{f}}{\rm{r}}}(\sqrt{{R}_{{\rm{P}}{\rm{P}}}}-\frac{{Q}_{{\rm{P}}{\rm{f}}{\rm{r}}}}{2})}{{R}_{{\rm{P}}{\rm{P}}}{(1+({Q}_{{\rm{P}}{\rm{f}}{\rm{r}}}-1){{\rm{e}}}^{-{Q}_{{\rm{P}}{\rm{f}}{\rm{r}}}{\gamma }_{Pr}t})}^{2}}$$

**Figure 4:**
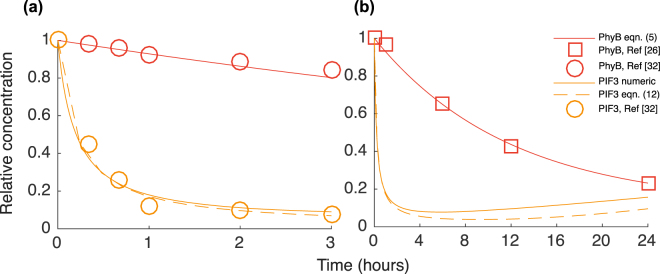
Calculated dynamic response of PhyB and PIF3 after light induction. In (**a**) the response for the first 3 hours and in (**b**) the response during the first 24 hours. In darkness (*t* < 0) there are no phytochromes in the Pfr form and the level of PIF3 is high. After light induction at *t* = 0 the phytochromes switch (approximately instantaneously) to the Pfr form. The faster decay of Pfr compared to Pr gives that the Pfr (and thus total PhyB) level decrease with time after the light-switch as is seen in both (**a**) and (**b**). The PIF3 concentration is initially decreasing rapidly since Pfr induce phosphorylation of PIF3 followed by degradation by 26S-proteasomes. Morover, since Pfr is also decreasing, the phosphorylation rate of PIF3 decrease and PIF3 will after the initial rapid decay slowly increase somewhat after the first day. As a reference for the calculated model data and for parameter estimates, we have in (**a**) inserted PhyB and PIF3 data (circles) from figure 7F in ref.^32^ and in (**b**) PhyB data (squares) extracted from Fig. 4C in ref.^26^.

Thus we now have an explicit solution of the PIF concentration. In Fig. 4 we display the calculated Pfr concentration, eqn. (5), and the PIF concentration, both from numerically integrating eqn. (8) and from the approximation given by eqn, (12). To obtain good estimations for the kinetic parameters of the calculated Pfr and PIF3 response we have in the figure inserted data of PhyB decay in red light (in which Pfr is dominating) extracted from two independent measurements, from figure 7F in ref.^32^. and in Fig. 4C in ref.^26^. Both data sets give us additional confirmation of the approximately 8 hours half-life reported in ref.^29^. In Fig. 4a we have inserted PIF3 response after light induction both calculated and measured, extracted from figure 7F in reference^32^. The half-life for PIF3 in red light is measured to be approximately 15 minutes. This is obtained approximately by setting $\mathrm{1/}{\gamma }_{{\rm{PIF}}}{R}_{{\rm{PP}}}$ to 15 minutes (assuming PhyB $\approx 1$ the first 15 minutes). Setting ${R}_{{\rm{PP}}}=350$ and ${Q}_{{\rm{Pfr}}}=8.75$ agree with PhyB and PIF3 measured decay in red light.

### PIF regulated nuclear components

Assume that nuclear gene *X* is under negative regulation of PIF, the synthesis of *X* is then a function of the PIF concentration ${\nu }_{{\rm{P}}{\rm{I}}{\rm{F}}}$. The concentration of *X* transcripts are then given by

13 $$\frac{d[X]}{dt}={\nu }_{{\rm{PIF}}}-\gamma [X]$$

where, as usual, *γ* is the decay rate constant of the molecule of interest, here *X*. If we assume fast binding and dissociation of the PIF repressor, compared to the transcription rate, then the probability of PIF being bound to the promoter of *X* can be expressed in terms of the PIF concentration. Assuming that PIF bind as a dimer, the transcript rate of ${\nu }_{{\rm{PIF}}}$ equals the maximal transcription rate ${V}_{{\rm{\max }}}$ multiplied with the probability that the promoter is not bound with PIF, which is given by

14 $${\nu }_{{\rm{P}}{\rm{I}}{\rm{F}}}(t)=\frac{{V}_{{\rm{m}}{\rm{a}}{\rm{x}}}}{1+{[{\rm{P}}{\rm{I}}{\rm{F}}]}^{2}/{K}_{{\rm{P}}{\rm{I}}{\rm{F}}}}$$

where ${K}_{{\rm{PIF}}}$ is the dissociation constant of PIF binding to the promoter of *X*. Now, let $[{\rm{PIF}}]$ denote the relative to dark concentration by a scaling of the dissociation constant ${K}_{{\rm{PIF}}}$ with the dark level concentration, ${K}_{{\rm{P}}{\rm{I}}{\rm{F}}}\mapsto {[{\rm{P}}{\rm{I}}{\rm{F}}]}_{{\rm{D}}}{K}_{{\rm{P}}{\rm{I}}{\rm{F}}}$. Combining (13), (14), with $[{\rm{PIF}}]=1$ in darkness and setting time derivative to zero gives the stationary dark concentration of *X* as

15 $$[{X}_{{\rm{D}}}]=\frac{{V}_{{\rm{m}}{\rm{a}}{\rm{x}}}}{\gamma }\frac{{K}_{{\rm{P}}{\rm{I}}{\rm{F}}}}{{K}_{{\rm{P}}{\rm{I}}{\rm{F}}}+1}$$

Since we are interested in the relative to dark stationary concentration of transcripts we can re-write equation in terms of the relative to dark concentration, implying that now $[X]\mapsto [X]/[{X}_{{\rm{D}}}]$. Using (13), (14) and (15) we obtain the evolution of the relative to dark *X* transcript level as a function of the (relative) PIF concentration as

16 $$\frac{d[X]}{dt}=\gamma (\varphi (t)-[X])$$

where

17 $$\varphi (t)=\frac{{K}_{{\rm{PIF}}}+1}{{K}_{{\rm{PIF}}}+{[{\rm{PIF}}]}^{2}(t)}$$

is the relative (to dark) synthesis rate of *X*. In darkness, where $[{\rm{PIF}}]=1$ and thus $\varphi (t)=1$, we obtain the initial (dark) concentration $[X]=1$ as desired. The time evolution $[X](t)$ after the light-switch is given by the integral equation

18 $$[X](t)={e}^{-\gamma t}+\gamma {\int }_{0}^{t}\varphi (s){e}^{-\gamma (t-s)}ds$$

The integrand is the product of the (relative) synthesis rate at time *s* and the degradation that will occur between *s* and *t*. The integral thus sums the amount of molecules that remain at time *t* that has been synthesized over the interval from 0 to *t*. In general the integral can not be evaluated directly to give a closed expression. For short times, $t\mathrm{ < 1/}\sqrt{{\gamma }_{{\rm{Pfr}}}\gamma }$ we can approximate the integral using eqn. (10) which gives that

19 $$[X](t)\approx (1+(\frac{{K}_{{\rm{P}}{\rm{I}}{\rm{F}}}+1}{{K}_{{\rm{P}}{\rm{I}}{\rm{F}}}})(\gamma t+\frac{{(\gamma t)}^{2}}{2})){e}^{-\gamma t}\,,\,\,\,t < 1/\sqrt{{\gamma }_{{\rm{P}}{\rm{f}}{\rm{r}}}\gamma }$$

The stationary solution can be computed directly from eqn. (13) by setting the left hand side to zero and inserting the stationary PIF solution. The stationary solution is approximately

20 $$[X](t\to \infty )\approx \frac{{K}_{{\rm{PIF}}}+1}{{K}_{{\rm{PIF}}}+{(\frac{{Q}_{{\rm{Pfr}}}}{\sqrt{{R}_{{\rm{PP}}}}}+\frac{{Q}_{{\rm{Pfr}}}^{2}}{2})}^{2}}$$

**Figure 5:**
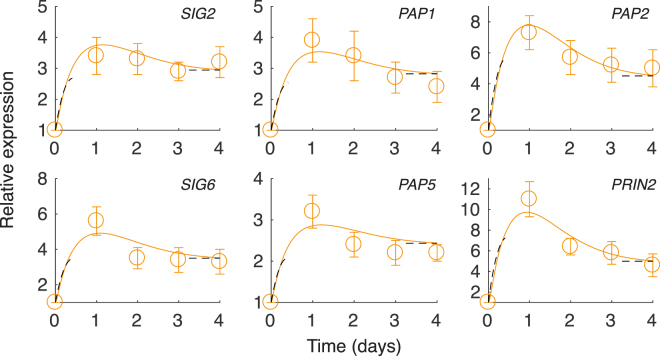
Model (lines) and experimental (circles) gene expression profiles of PEP associated nuclear genes (SIGs, PAPs and PRIN2). 7-day-old cells cultured in MS + 3% sucrose in dark were sub-cultured in MS + 1% sucrose and equilibrated before being shifted to under constant light at day zero. The constant light remain throughout the experiment and have a R/FR ratio of 10:1. The panels display the relative gene expression from real-time PCR in light relative to dark level at day zero, where both the light and dark expressions are normalized to expression of the gene encoding ubiquitin like protein (At4g36800). Each data point represents the mean (±SE) of at least 9 independent experiments. The model data is calculated by numerically integrating equation 16. For each calculated expression profile the unknown parameter *K*_PIF_, the relative dissociation rate constant of PIF to its binding site, is set to match the measured expression profile.The black dashed lines are the short-time approximation, eqn. (19) for $t < 1/\sqrt{{\gamma }_{{\rm{Pfr}}}\gamma }$, and for long times the stationary solution, eqn. (20).

In Fig. 5 we display the calculated and measured relative (to dark) expression for a number nuclear genes that have PIF3 binding motifs in the promoter; *SIG*s, *PAP*s and *PRIN2*. The genes display a similar profile with an initial sharp response with a more or less pronounced peak during the first day which is followed by a slower decay. The sharp increase is due to the rapid shift from Pr to the active Pfr that induce a rapid degradation of PIF3 and thus a sharp increase in the expression of PIF3 repressed genes. The rate of increase and the height of the peak is dependent on the relative dissociation rate constant, a behaviour that is captured by the short-time approximation (19)–relatively stronger binding (small *K*_PIF_) of PIF3 gives an initial more rapid increase and a more distinct peak and vice versa. The following decay in the gene expression after the initial high peak during the first day can be explained by the decreasing amount of Pfr with time, making the PIFs less frequently phosphorylated and decayed by the 26 S proteasomes. As a result, the PIF3 increase slightly after the initial low value during the first day and the expression of genes repressed by PIF3 will decrease. The short-time approximation eqn. (19) for $t\mathrm{ < 1/}\sqrt{{\gamma }_{{\rm{Pfr}}}\gamma }$ and the stationary solution eqn. (20) are both inserted as black dashed lines. The half-life of a large number of mRNA molecules in *A. thaliana* were measured in^33^ giving half-lives spanning from approximately 0.2 to 12 hours and with the mean mRNA half-life of 6 hours. Since we do not know the exact values of our nuclear transcripts, we will for simplicity set the decay rates of all nuclear transcripts to 6 hours. In this way we only need to set one parameter, the dissociation rate constant *K*_PIF_, for each of the gene expression profiles. The decay rate of the different transcripts may of course vary, but the upside of such a simplification is that the individual behaviour now can be explained by the difference in the repressor strength of PIF3. By setting the (relative) dissociation rate constant of PIF3, *K*_PIF_ =  0.32, 0.22, 0.35, 0.12, 0.48 and 0.09 for *SIG2*, *SIG6*, *PAP1*, *PAP2*, *PAP5* and *PRIN2*, respectively, we can make the model data fall within the error for most of the measured data. To highlight the effect of *phyB* inhibited mutants we have modelled the expression of SIG2 and SIG6 by scaling the PhyB levels by a factor of *q*. For *q* < 1 the levels of PhyB is decreased and the phosphorylation rate and removal of PIF3 is slower and the repression of SIGs and PAPs will increase. For $q\approx \mathrm{1/4}$ the model data match the experimentally measured expression levels of SIG2 and SIG6. The results are displayed in Supplementary Fig. S1.

## Discussion

Photosynthesis provides the energy and reduced carbon required for practically all life on our planet, as well as the molecular oxygen necessary for our survival. In plants and algae photosynthesis occurs in chloroplasts that are only a few microns in size. Although performed in a tiny compartment, the photosynthetic process has a profound impact on our atmosphere and climate. When plastids of eukaryotic cells differentiate into a photosynthetically active chloroplast, the process follows a clear developmental program. All chloroplasts are derived from non-photosynthetic progenitors, either directly from proplastids present in meristematic cells, or via the dark-grown intermediate form known as etioplasts. The chloroplasts have their own genome, but throughout the evolution many genes have been transferred to the nuclear genome. Since proplastids can be developed to attain different cellular functions and since the photosynthetic machinery is localized to chloroplasts but built using proteins encoded both in the nucleus and in the plastids the cells require a control system that coordinate the activities of these two distinct genomes.

PIFs are basic helix-loop-helix transcriptional regulators that interact with the active Pfr form of Phy, and regulators of photomorphogenic development. The mechanistic action of PIFs is believed to be dual where PIF3 was suggested to act both as a negative regulator and a positive regulator of light response. However, regarding chloroplast development the current consensus is that PIFs implement an etiolated program of growth (skotomorphogenesis) including elongation of hypocotyls, maintenance of non-photosynthetic cotyledons and repression of chloroplast development^13,34,35^. Once the seedlings are exposed to light, the phytochromes rapidly switch to the active form and phosphorylates PIFs which is subsequently degraded by the 26S-proteasome pathway. As a consequence of PIF degradation upon the switch from dark to light, the seedlings exit the etiolated state^34^; hypocotyl growth is no longer accelerated and photomorphogenesis is released from repression.

We find that expression of the components required for chloroplast development, the essential PEP components; SIGs, PRIN2 and PAPs, are rapidly induced upon light exposure and display a peak of cellular mRNA levels within the first 24 hours, a peak which is 3–10 times higher compared to dark levels. Our model suggests that expression of the nuclear encoded components, *SIG*s and *PAP*s, is initiated by the light activation of the PhyB-PIF3 pathway. The model is based on the following data; first, *SIG*s and *PAP*s all contain PIF3 binding motifs non-randomly distributed in their promoters, secondly, the expression profile of *SIG2* and *SIG6* throughout the initial light response only deviate from the wild-type expression profile in the *phyB* mutant when *cry1cry2*, *phyA* and *phyB* mutants were investigated. Finally, the expression profiles of *SIG*s and *PAP*s follow the model predicted expression profile in our theoretical model of a gene repressed by PIF3 in the dark. All components display the same generic behaviour anticipated by the model calculations–an initial fast accumulation of mRNA levels as a response to light which is shortly reaching a maximum and followed by a slower decay. The model includes the kinetic components as well as the interactions that lead to the transcriptional response of the studied genes and can be exploited to analyse the effect of different molecular or genetic perturbations. However, in the combined ChIP-seq and RNA-seq experiment performed to identify PIF3 dependent genes, the genes encoding SIGs and PAPs did not qualify for the list of 22 genes assigned as direct-targets of PIF3^12^. The criteria used were strict and given the phenotype of the *pif3* mutant it is possible that many more genes than 22 are direct targets of PIF3. In this experiment whole two-day-old dark-grown seedlings were used, which do not provide a true reflection of what occurs in the developing leaves of a plant since chloroplast development proceeds differently in cotyledons and true leaves^36^. Moreover, the chromatin structure may prevent binding of transcription factors in specific regions of the genome at specific stages in the cell development and as a consequence ChiP-seq using many different cell types may result in weak signals for many of the binding locations^37,38^. Therefore, performing ChIP-seq experiments using a cell culture behaving like leaf mesophyll cells, additional PBE- and G-box binding targets of PIF3 accessible during photomorphogenesis may display ChiP-seq enrichment peaks.

Even though the model we formulate is a simplification it seems to capture the basic ingredients of how the light signal is transferred biochemically to the level of gene expression. The model approach provides analytical expressions with scaled parameters that describe the regulatory kinetics for a range of situations of light compositions, phosphorylation rates and equilibrium constants. A more elaborate model would lead to computer simulations, while being more accurate, typically only describing the behaviour of the system for a limited number of parameter values. The model quantifies the generic light response of a gene repressed by PIF3 in the dark. Therefore, when chloroplast development progress in response to light, the quantified response can be used to investigate genes that are under direct control of PIF3 and further to explore deviations from the expected behaviour to identify additional regulatory components in the development. For alternating dark and light conditions the model maybe need to include additional states of PhyB, such as e.g. nuclear bodies, in case a simple one-step dark reversion reaction of Pfr to Pr does not capture the kinetics during consecutive switches between dark and light. Using a mathematical model leading to explicit equations we have shown how the activities of two distinct genomes can be coordinated by exploiting a two-state molecular switch to induce transcriptional re-programming of the participating components. Every eukaryotic cell has at least two genomes, thus the mechanisms enabling the synchronization of these genomes is of great general interest. Consequently, the approach presented to the readers can be used to address similar questions within fundamental biology.

## Materials and Methods

### Cell culture and growth conditions

Arabidopsis thaliana (Col-0) cell lines were grown in Murashige and Skoog (MS) medium supplied with 3% (w/v) sucrose, pH 5.7 in the dark at 25 °C, shaken at 140 rpm^16^. For all experiments, 7-day-old cells from dark conditions were subcultured in a 1:10 ratio in MS medium 1% (w/v) sucrose, equilibrated and placed in a growth cabinet under continuous light (150 *μ*mol photons m-2 s-1) and constant rotary agitation. The arabidopsis seedlings were grown on 1× MS plates. The mutants are described in^39^. To test the effect of sucrose concentrations, seedlings are grown both in 1% and 0% sucrose, see Supplementary Fig. S1.

### Chlorophyll content analysis

Chlorophyll was extracted by adding 1 ml of buffered acetone (80% acetone, 0.2 M TrisHCl pH 7.0) to 80 mg FW of cells. Samples were incubated overnight at 4 °C and centrifuged for 10 min at 14000 rpm. Chlorophyll content was measured and calculated according to ref.^40^.

### Confocal and transmission electron microscopy

Analysis of chlorophyll autofluorescence was performed by confocal laser scanning microscopy (Zeiss LSM 780). The cell wall was stained by incubating samples with calcofluor-white (0.002% final concentration). Cells were scanned sequentially to prevent any crosstalk between fluorescence channels. For transmission electron microscopy, the samples were prepared according to ref.^19^.

### RNA isolation and real-time PCR

Total RNA was isolated using the EZNA plant RNA kit (Omega biotek) according to the manufacturer’s instructions. After incubation with RNase-free DNase I (Thermo scientific), 0.5 *μ*g of RNA was reversed transcribed into cDNA using the iScript cDNA synthesis kit (Bio-Rad) according to the manufacturer’s instructions. Gene expression was analyzed with 3 *μ*l of 10-fold diluted cDNA in 10 *μ* iQ SYBR Green Supermix reaction (Bio-Rad) on a CFX96 real-time PCR detection system (Bio-Rad).

### MEME analysis

Promoter sequences within 3 kb of the translation start site for the SIG and PAP genes were extracted from TAIR

(www.arabidopsis.org) and the tool “Download upstream sequences” and subjected to MEME-FIMO^41^

(http://meme-suite.org/tools/fimo) to scan for G-box (CACGTG) and PBE-box (CACATG) motifs at both strands.

## Electronic supplementary material

Supplementary Information
