# Supplementary material for: A quantitative model of the phytochrome-PIF light signalling initiating chloroplast development
Source: Sci Rep. 2017 Oct 24;7:13884. doi: 10.1038/s41598-017-13473-2 (PMC5655028; doi:10.1038/s41598-017-13473-2)
Supplement: Supplementary file 1 — Supplementary Information [file 41598_2017_13473_MOESM1_ESM.pdf]

Supplementary information:
A quantitative model of the phytochrome-PIF light signalling initiating chloroplast development

Carole Dubreuil[1], Yan Ji[1], Åsa Strand[1], and Andreas Grönlund[1,a]

[1] Umeå Plant Science Centre, Department of Plant Physiology, Umeå University, SE-90187 Umeå, Sweden
[a] andreas.gronlund@umu.se

Supplementary figures

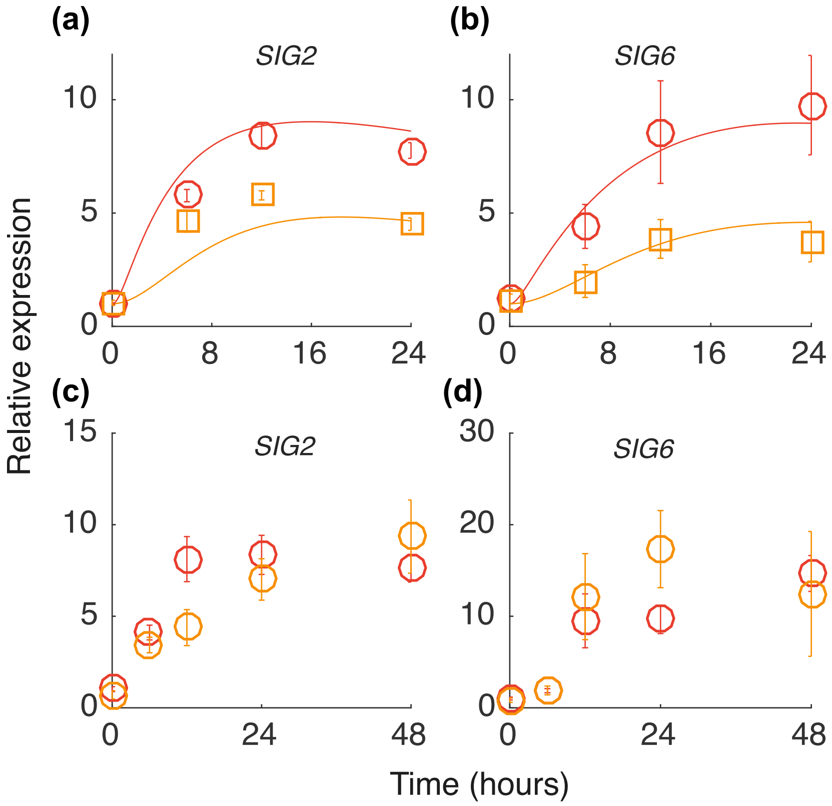

Supplementary Figure S1. Mutant and sucrose expression profiles. Expression after light induction of SIG2 (a) and SIG6 (b) in WT (red circle symbols) and *phyB* mutant (orange square symbols). Model data WT (red lines) and where PhyB levels are reduced with a factor 1/4 (orange lines). For *SIG2* the mRNA half-life were set to 3 hours and for *SIG6* 6 hours. In (c) and (d) expression after light induction using 1% sucrose (red) and 0% sucrose (orange). 5-day old dark grown seedlings were exposed to light and sampled for RNA-extraction.

Supplementary tables

Supplementary Table S1

MEME-FIMO analysis 3 kb upstream of ATG

**G-box**				**Distance from 3 kb upstream**			
	**Motif**	**Sequence Name**	**Strand**	**Start**	**End**	**p-value**	**Matched Sequence**
pTAC3/PAP1	1	AT3G04260	+	429	434	0.000195	CACGTG
pTAC3/PAP1	1	AT3G04260	+	2887	2892	0.000195	CACGTG
pTAC2/PAP2	1	AT1G74850	+	1556	1561	0.000195	CACGTG
pTAC2/PAP2	1	AT1G74850	+	2728	2733	0.000195	CACGTG
MurE/PAP11	1	AT1G63680	+	1457	1462	0.000195	CACGTG
PRIN2	1	AT1G10522	+	2502	2507	0.000195	CACGTG
PRIN2	1	AT1G10522	-	2502	2507	0.000195	CACGTG
SIG4	1	AT5G13730	+	1713	1718	0.000195	CACGTG
SIG4	1	AT5G13730	+	2518	2523	0.000195	CACGTG
SIG5	1	AT5G24120	+	1201	1206	0.000195	CACGTG

Supplementary Table S2

MEME-FIMO analysis 3 kb upstream of ATG

**PBE-box**				**Distance from 3 kb upstream**			
	**Motif**	**Sequence Name**	**Strand**	**Start**	**End**	**p-value**	**Matched Sequence**
pTAC3/PAP1	1	AT3G04260	+	2550	2555	0.000237	CACATG
pTAC3/PAP1	1	AT3G04260	+	2660	2665	0.000237	CACATG
pTAC3/PAP1	1	AT3G04260	+	2898	2903	0.000237	CACATG
pTAC10/PAP3	1	AT3G48500	-	2253	2258	0.000237	CACATG
pTAC10/PAP3	1	AT3G48500	-	2618	2623	0.000237	CACATG
pTAC10/PAP3	1	AT3G48500	-	2706	2711	0.000237	CACATG
pTAC10/PAP3	1	AT3G48500	+	1369	1374	0.000237	CACATG
pTAC12/PAP5	1	AT2G34640	-	1281	1286	0.000237	CACATG
pTAC12/PAP5	1	AT2G34640	+	645	650	0.000237	CACATG
pTAC12/PAP5	1	AT2G34640	+	1222	1227	0.000237	CACATG
pTAC12/PAP5	1	AT2G34640	+	2335	2340	0.000237	CACATG
FLN1/PAP6	1	AT1G69200	-	573	578	0.000237	CACATG
FLN1/PAP6	1	AT1G69200	+	1398	1403	0.000237	CACATG
FLN2	1	AT3G54090	-	1427	1432	0.000237	CACATG
MurE/PAP11	1	AT1G63680	-	272	277	0.000237	CACATG
MurE/PAP11	1	AT1G63680	-	320	325	0.000237	CACATG
MurE/PAP11	1	AT1G63680	-	1446	1451	0.000237	CACATG
MurE/PAP11	1	AT1G63680	-	1585	1590	0.000237	CACATG
MurE/PAP11	1	AT1G63680	+	67	72	0.000237	CACATG
PRIN2	1	AT1G10522	-	1500	1505	0.000237	CACATG
PRIN2	1	AT1G10522	+	557	562	0.000237	CACATG
SIG1	1	AT1G64860	+	4	9	0.000237	CACATG
SIG2	1	AT1G08540	+	1570	1575	0.000237	CACATG
SIG3	1	AT3G53920	-	1348	1353	0.000237	CACATG
SIG3	1	AT3G53920	+	1346	1351	0.000237	CACATG
SIG3	1	AT3G53920	+	2127	2132	0.000237	CACATG
SIG4	1	AT5G13730	-	2576	2581	0.000237	CACATG
SIG4	1	AT5G13730	+	664	669	0.000237	CACATG
SIG4	1	AT5G13730	+	1377	1382	0.000237	CACATG
SIG4	1	AT5G13730	+	1571	1576	0.000237	CACATG
SIG5	1	AT5G24120	+	2181	2186	0.000237	CACATG
SIG6	1	AT2G36990	+	265	270	0.000237	CACATG

Supplementary Table S3
MEME-FIMO analysis 3 kb upstream of ATG

PBE-box core			Distance from 3 kb upstream				
	**Sequence Name**	**Strand**	**Start**	**End**	**p-value**	**q-value**	**Matched Sequence**
**FSD3/PAP4**	AT5G23310	-	690	695	0.00298	0.951	AACATG
		-	1351	1356	0.00298	0.951	AACATG
		-	1566	1571	0.00298	0.951	AACATG
		+	575	580	0.00298	0.951	AACATG
		+	688	693	0.00298	0.951	AACATG
		-	1074	1079	0.00456	0.951	CACATT
		-	2347	2352	0.00456	0.951	CACATT
		+	259	264	0.00346	0.951	GACATG
**pTAC14/PAP7**	AT4G20130	+	2057	2062	0,00298	0,951	CACATA
		+	1471	1476	0,00369	0,951	CACATC
		+	1476	1481	0,00369	0,951	CACATC
		-	1541	1546	0,00456	0,951	CACATT
		+	625	630	0,00456	0,951	CACATT
		+	1607	1612	0,00456	0,951	CACATT
		+	1699	1704	0,00456	0,951	CACATT
		+	2358	2363	0,00456	0,951	CACATT
		-	2557	2562	0,00346	0,951	GACATG
		-	1678	1683	0,00456	0,951	TACATG
		+	2531	2536	0,00456	0,951	TACATG
**pTAC6/PAP8**	AT1G21600	-	1385	1390	0.00298	0.951	AACATG
		+	1265	1270	0.00298	0.951	AACATG
		-	2038	2043	0.00298	0.951	CACATA
		+	1183	1188	0.00298	0.951	CACATA
		-	1630	1635	0.00369	0.951	CACATC
		+	649	654	0.00369	0.951	CACATC
		+	1345	1350	0.00369	0.951	CACATC
		-	1678	1683	0.00456	0.951	CACATT
		+	114	119	0.00456	0.951	CACATT
		+	178	183	0.00456	0.951	CACATT
		+	2086	2091	0.00456	0.951	CACATT
		+	1250	1255	0.00346	0.951	GACATG
**FSD2/PAP9**	AT5G51100	+	456	461	0.00298	0.951	AACATG
		+	913	918	0.00298	0.951	AACATG
		-	8	13	0.00298	0.951	CACATA
		-	368	373	0.00298	0.951	CACATA
		+	2150	2155	0.00298	0.951	CACATA
		+	2671	2676	0.00298	0.951	CACATA
		-	1464	1469	0.00369	0.951	CACATC
		+	1200	1205	0.00456	0.951	CACATT
		+	2628	2633	0.00456	0.951	CACATT
		-	404	409	0.00346	0.951	GACATG
		-	458	463	0.00346	0.951	GACATG
		+	1000	1005	0.00346	0.951	GACATG
		-	2745	2750	0.00456	0.951	TACATG
		+	402	407	0.00456	0.951	TACATG
**TrxZ/PAP10**	AT3G06730	-	518	523	0.00298	0.951	AACATG
		+	1042	1047	0.00298	0.951	AACATG
		+	1314	1319	0.00298	0.951	AACATG
		+	2332	2337	0.00298	0.951	AACATG
		-	1157	1162	0.00298	0.951	CACATA
		-	325	330	0.00369	0.951	CACATC
		+	736	741	0.00456	0.951	CACATT
		+	910	915	0.00456	0.951	CACATT
		-	723	728	0.00346	0.951	GACATG
		-	1044	1049	0.00346	0.951	GACATG
		+	2041	2046	0.00346	0.951	GACATG
**pTAC7/PAP12**	AT5G24314	-	60	65	0.00298	0.951	AACATG
		+	2297	2302	0.00298	0.951	AACATG
		-	512	517	0.00298	0.951	CACATA
		-	1664	1669	0.00298	0.951	CACATA
		-	181	186	0.00369	0.951	CACATC
		-	531	536	0.00369	0.951	CACATC
		+	1210	1215	0.00369	0.951	CACATC
		-	1992	1997	0.00456	0.951	CACATT
		+	58	63	0.00456	0.951	TACATG
		+	544	549	0.00456	0.951	TACATG
